# Research progress of PBX1 in developmental and regenerative medicine

**DOI:** 10.7150/ijms.80262

**Published:** 2023-01-22

**Authors:** Hao Chen, Zhuyuan Yu, Ye Niu, Litian Wang, Kan Xu, Jinyu Liu

**Affiliations:** 1 Department of Neurovascular Surgery, First Hospital of Jilin University, 1 Xinmin Avenue Changchun 130021, Jilin Province, China.; 2 Department of Toxicology, School of Public Health, Jilin University, Changchun 130021, Jilin Province, China.

**Keywords:** Pbx1, Development, Regenerative medicine, Transcription factors

## Abstract

Pre-B-cell leukemia transcription factor 1 (PBX1) proteins are a subfamily of evolutionarily conserved atypical homeodomain transcription factors belonging to the superfamily of triple amino acid loop extension homeodomain proteins. PBX family members play crucial roles in the regulation of various pathophysiological processes. This article reviews the research progress on PBX1 in terms of structure, developmental function, and regenerative medicine. The potential mechanisms of development and research targets in regenerative medicine are also summarized. It also suggests a possible link between PBX1 in the two domains, which is expected to open up a new field for future exploration of cell homeostasis, as well as the regulation of endogenous danger signals. This would provide a new target for the study of diseases in various systems.

## Introduction

Congenital genetic defects, autoimmune diseases, diabetes, neurodegenerative disorders, and trauma all involve the loss or dysfunction of healthy human tissue and contribute greatly to human disability. Some improvements in clinical management of symptoms and slowing of disease progression have been made for some of these conditions, but restorative and regenerative therapies are still severely lacking. Stem cells possess the unique ability to self-renew and differentiate and play vital roles in embryonic development and tissue and organ regeneration; as such, they are attractive candidates for cell therapy and regenerative medicine 1-4. Stem cells maintain their unique properties through several mechanisms including pluripotent transcription factors 5, signaling pathways 6, specific microRNAs 7, long non-coding RNAs 8, alternative pre-messenger RNA splicing 9, histone modification 10, and cell cycle progression 11. These mechanisms play key roles in embryonic development and tissue and organ regeneration.

Pre-B-cell leukemia transcription factor 1 (PBX1) is a member of the triple amino acid loop extension family of homologous transcription factors 12. It is well known for its function in lymphocytic leukemia 13 and several cancers 14-20. PBX1 also plays an important role in regulating stem cell developmental gene expression 21, maintaining stem cell stemness and self-renewal 22, 23, and regulating cellular oxidative stress and apoptosis during development 24. It also plays a role in anti-aging, tissue and organ homeostasis, and tissue and organ regeneration 25-27. Thus, PBX1 is a promising therapeutic target and biomarker in developmental biology and regenerative medicine.

In this review, we summarize the structure of PBX1, its regulatory mechanisms involved in the development of various tissues and organs, and research targets of PBX1 in regenerative medicine. A possible relationship between PBX1 in these two fields is proposed, which is expected to open up a new perspective for exploring cell homeostasis and tissue and organ reconstruction in the future.

## PBX1 structure, development, and function in regenerative medicine

### PBX1 structure

PBX1 is approximately 430 amino acid residues long, missing 78 amino acid residues in the N-terminal domain and a 30-residue stretch in the C-terminal domain, and contains a 60-residue-long homeodomain (HD) that mediates its binding to DNA or other proteins 28. The HD and C termini of PBX proteins can also mediate their interaction with Hox/HOM-C proteins, thus playing a role in transcription 29, 30. PBX proteins comprise two highly conserved protein-protein interaction domains, including PBC-A and PBC-B 31. The PBC-A domain of PBX members can mediate interactions with PREP/MEIS proteins to form a transcription regulatory complex that plays a role in development 32. The PBC-A domain also mediates the interaction of PBX1 with CRM1, which is required for the nuclear export of PBX1, while the PBC-B domain in PBX1 can be specifically phosphorylated by PKA, leading to PBX1 accumulation in the nucleus 33. In addition, NMHCB competes with the PBC-B region of PBX1 for interaction, leading to cytoplasmic accumulation of PBX1 34.

### Development

PBX1 plays an important role in tissue and organ development by enhancing the recruitment of homeobox genes, enhancing the expression of developmental homeobox genes, and promoting cell proliferation and differentiation through epigenetic regulation. Moreover, genes regulating oxidative stress and apoptosis also promote cell development (FURGE1).

#### PBX1 enhances the recruitment of homeobox genes and the expression of target genes that regulate cellular proliferation and differentiation

In fact, PBX1 is a key transcriptional regulator of the development of multiple tissues and organs. Its tight binding to homeobox genes facilitates their transcriptional regulation. In the nervous system, PBX1 and its family members are important transcriptional regulators of neurons 35-38. For instance, Hoxc5 affects many aspects of motor neuron (MN) development and function 39-41. In D2 MN cell cultures, at shared binding sites, it showed higher binding to Pbx1, and the possible mechanism is that PBX1a enhances the recruitment of Meis1 to increase Hoxc5 expression 42. Another study indicated that PBX1 interactions with HOX proteins and DNA are dispensable for the RA-induced ability of ES cells to express neural genes 43. In the adult mammalian olfactory bulb, the transcription factor PBX1 controls neurogenesis in progenitor cells and the survival of migrating neuroblasts. Furthermore, PBX1 acts downstream or in parallel with other transcription factors such as Dlx2, Pax6, Etv1, Meis2, and Couptf1 that are required for dopaminergic specification of neurons. During midbrain dopaminergic neuron development, PBX1a is present in developing mDA and type 2 neuroblasts and improves the differentiation of neural progenitor cells towards mDA fate. The molecular mechanism by which PBX1 regulates mDA development may be that PBX1 controls the specification of mDA neurons by directly activating Pitx3 and repressing lateral fate genes, such as Onecut2. Moreover, by combining with recruited development-related genes, it promotes the expression of target genes and promotes the development of tissues and organs. For instance, early expression of PBX1a promotes the expression of neuronal genes, such as Phox2b, Cntn2, Ntng2, Olig1, Isl1, Nrp2, Ngfr, Nav2, Slit1, Igf2, Dlk1, and Meox1 17, 21, 42-46.

In addition to neuronal differentiation, PBX1 is crucial for the proper differentiation of stem cells and tissue development in other organs. Binding of PBX1 to Meis1 and Rnux1 during the morphogenesis of the diaphragm and lung branches is also crucial, and its reduced expression disrupts lung mesenchymal cell proliferation and proper airway branch development 47. PBX1 is also an important epigenetic regulator of the balance between pulmonary vasoconstriction and vasodilation. Studies have shown that, several genes that promote VSM contraction (endothelin-1 [Edn1], angiotensinogen [Agt], and smooth muscle myosin [Myh11]) were upregulated, whereas genes that promote VSM relaxation (natriuretic peptide C [Nppc] and adenylate cyclase 8 [Adcy8]) were downregulated in PBX1 mutants. Interestingly, both Edn1 and Agt gene promoters contain conserved PBX binding consensus elements: binding elements within the transcriptional regulatory regions of the genome 48. Hox11 is a direct *in vivo* target of PBX1 and autoregulates its own promoter with PBX1 during spleen ontogeny. PBX1 and Hox11 genetically interact in spleen formation, and the loss of either is associated with a similar reduction in progenitor cell proliferation and failed expansion of the splenic anlage 49.

#### PBX1 participates in epigenetic regulation of transcription to maintain stem cell stemness and self-renewal during development

PBX is also involved in transcription regulation through histone modifications 50. MiR-181a-5p exerts its ossification function by downregulating the expression of PBX1. PBX1 has also been found to be an important negative regulator of ligament cell ossification. This molecular mechanism is partly due to PBX1- mediated modifications of H3K9me2 and H3K9ac in the promoter regions of genes related to ossification, such as OSX and OCN 51, 52. Moreover, in a study on hematopoietic stem cells, it was found that MiR-127-3p is absent in HSC obtained from PBX1-cKO mice, which display a profound self-renewal defect. These results suggest that PBX1 and its homeobox partners regulate the expression of miR-127-3p 53. In a study of ES cells, PBX1 was one of the genes whose Alternative splicing (ANS) was positively correlated with H3K36me3. They found that the transcription of two isoforms of PBX1, PBX1a and PBX1b, is regulated by H3K36me3 during hESC differentiation. Their protein isoforms competitively bind NANOG, and the binding of PBX1b abolishes the binding of PBX1a, further attenuating the activity of the core pluripotency regulatory network composed of Yamanaka factors 54. PBX1 splicing is dynamically regulated during olfactory bulb neurogenesis, with all dopaminergic cells expressing PBX1a but not PBX1b. Moreover, almost all circulating progenitor cells of the SEZ express PBX1b and lack the subtype expression of PBX1a 55. More research is needed to fully delineate the unique roles between these two different splice isoforms.

#### PBX1 regulates oxidative stress and apoptosis during development

Nfe2l1 is expressed during tyrosine hydroxylase (TH) neuron development at E12.5 24. It has been proposed that PBX1 directly controls the expression of the antioxidant transcription factor Nfe2l1, which counteracts oxidative stress, mitochondrial dysfunction, and proteasome damage 56-58. This promotes the survival of mDAn. Characterization of the cranial neural crest cell (CNCC) mutants revealed reduced proliferation of palatal progenitor cells, disturbed skeletal differentiation, and heterotopic ossification. This suggests a role of PBX1 in coordinating CNCC-dependent hypomorphological and skeletal differentiation 59. Knockdown of uc40-siRNA44 increases the number of cardiac stem cells during P19 cell differentiation. It promotes the proliferation of P19 cells and inhibits apoptosis induced by serum starvation, which may play a role in regulating PBX1 60.

PBX1 plays a critical role in the development of various tissues and organs. In addition, some sensitive targets have been identified in these studies. It also provides a direction for the treatment of clinical diseases. Each PBX1 isoform has different effects on development. Therefore, further studies on the modification and phenotype of PBX1 isoform switching are required.

### Regenerative medicine

#### Anti-aging: Inhibiting oxidative stress and reducing DNA damage to slow down stem cell aging

PBX1 is important for maintaining stem cell function by alleviating oxidative stress and preventing cellular apoptosis. In a hair follicle stem cell (HFSCS) study, PBX1 overexpression attenuated HFMSC senescence and apoptosis by alleviating reactive oxygen species (ROS) - mediated DNA damage without enhancing DNA repair. It has been found that PBX1 is an upstream regulator of PARP1 25. Further studies on HF-MSCs showed that PBX1 overexpression alleviated senescence and apoptosis of HF-MSC accompanied by up-regulation of SIRT1, down-regulation of PARP1, and increased intracellular NAD and ATP levels. SIRT1 knockdown enhanced cellular senescence and apoptosis, accompanied by increased ROS accumulation, aggravated DNA damage, and decreased intracellular NAD and ATP levels. PBX1 overexpression rescued HF-MSCs senescence and apoptosis induced by SIRT1 knockdown. PBX1 rescued ATP and NAD depletion mediated by PARP1 overexpression, accompanied by increased SIRT1 expression. This highlights a critical role of the PBX1-SIRT1-PARP1 axis in alleviating senescence and apoptosis in HF-MSCs 61.

PBX1 promotes stem cell reprogramming of hF-MSC into HF-iPSCs through epigenetic upregulation of NANOG. Specifically, PBX1 increased the efficiency of reprogramming by activating NANOG's promoter to up-regulate NANOG transcription and promote the expression of endogenous SOX2 and OCT4 62.

#### Maintenance of tissue and organ homeostasis

##### PBX1 regulates stem cell proliferation and cell cycle to maintain cell homeostasis

The proliferation of normal cells is tightly controlled and influenced by many signaling pathways to maintain homeostasis 63. PBX1 is usually associated with tumor growth by promoting proliferation and regulating cell cycle 19, 64- 67. However, many studies have shown that PBX1 also plays a crucial role in the proliferation of non-tumor stem cells 26, 62, 68, 69. Lichtenauer et al. demonstrated that PBX1, in addition to its role in genitourinary development and adrenocortical cells, is also required for the maintenance of adult adrenal growth, and *in vitro* studies have revealed that PBX1 and Sf-1 synergistically stimulates Mc2-r promoter activity. 26. In a study on hair follicle mesenchymal stem cells, Jiang et al. showed that PBX homeobox 1 enhances hair follicle mesenchymal stem cell proliferation through the AKT/glycogen synthase kinase signaling pathway and suppression of apoptosis. Moreover, ectopic expression of NANOG significantly upregulates PBX1 and results in decreased expression of p16 and p21 62.

##### PBX1 regulates the immune response during apoptosis to maintain cell homeostasis

PBX1 is a physiologically critical mediator of IL-10 gene transcription induced by apoptotic cells. In studies on the induction of IL-10 production, CD36 was found to be physiologically important, but not the only receptor for IL-10 induction by apoptotic cells. Further studies showed that PBX1 had little effect on basal IL-10 transcription in the absence of apoptotic cell stimulation. apoptotic cell-induced IL-10 production was significantly reduced. Therefore, the transcriptional potential of PBX1 highly depends on cell fate status and apoptotic cell signaling. Further research showed that PBX1b (PBX1a is not expressed in macrophages) is a major physiological and selective transcriptional mediator of apoptotic cell-induced IL-10 gene expression through ACRE 70.

#### Tissue regeneration

##### Nerve regeneration

PBX1 plays important roles in neurogenesis, the generation of new adult neurons from neural stem cells. In the nervous system, PBX1 plays an important role in the generation, survival, and terminal differentiation of adult V-SVZ neurons, and the deletion of transient amplifying progenitors (TAPs) can guide them from neurogenic to oligodendrogliogenic fate. Deletion of differentiating dopaminergic neurons disturbs their maturation and the expression of characteristic dopamine pathway genes 55, 71. Of note, PBX1 is important for the differentiation of neural progenitor cells towards a mDA fate 24. In fact, the neuron-specific gene doublecortin (Dcx) and the dopaminergic neuron marker gene tyrosine hydroxylase (Th) are known to be direct PBX1 target genes 72. Neuroblasts leave the SVZ and migrate into the olfactory bulb, where they eventually differentiate into different types of interneurons that are constantly replaced in existing circuits as part of lifelong remodeling of the mammalian olfactory system. Many transcription factors, including DLX2 and PAX6, bias progenitor cells toward a general neuronal fate and promote their subsequent maturation into specific types of interneurons 73. However, PBX1 can elicit the antioxidant effect of NFE2L1 on TH+ neurons, and lentiviral overexpression of PBX1 in human NES cells differentiated into mDAn significantly reduced the number of TH+ and aCASP3+ cells in cultures treated with 100 µmol H2O2 24, 74.

##### Angiogenesis

PBX1 also plays important roles in angiogenesis. In adult tissues, angiogenesis occurs transiently and in a highly regulated manner in events such as wound healing, tissue remodeling, and the female reproductive cycle 63. Of note, PBX1b is required for proangiogenic Hox DNA binding and transcriptional activity in endothelial cells. It was observed that endothelial cells (EC) predominantly express the Pbx1b isoform. Nuclear extracts of angiogenic EC expressed higher levels of active PBX1 on PBX1/Hox consensus DNA oligos and formed complexes more efficiently than nuclear extracts of quiescent ECs did. Furthermore, In the absence of PBX1, Hox D3 fails to induce the expression of integrin avb3, a key angiogenic mediator, and endothelial cells fail to migrate and form new blood vessels as a result 27. Thus, PBX1 may serve as an important therapeutic target or biomarker for diseases requiring angiogenic modulation, such as stroke and cancer.

**Figure 2:**
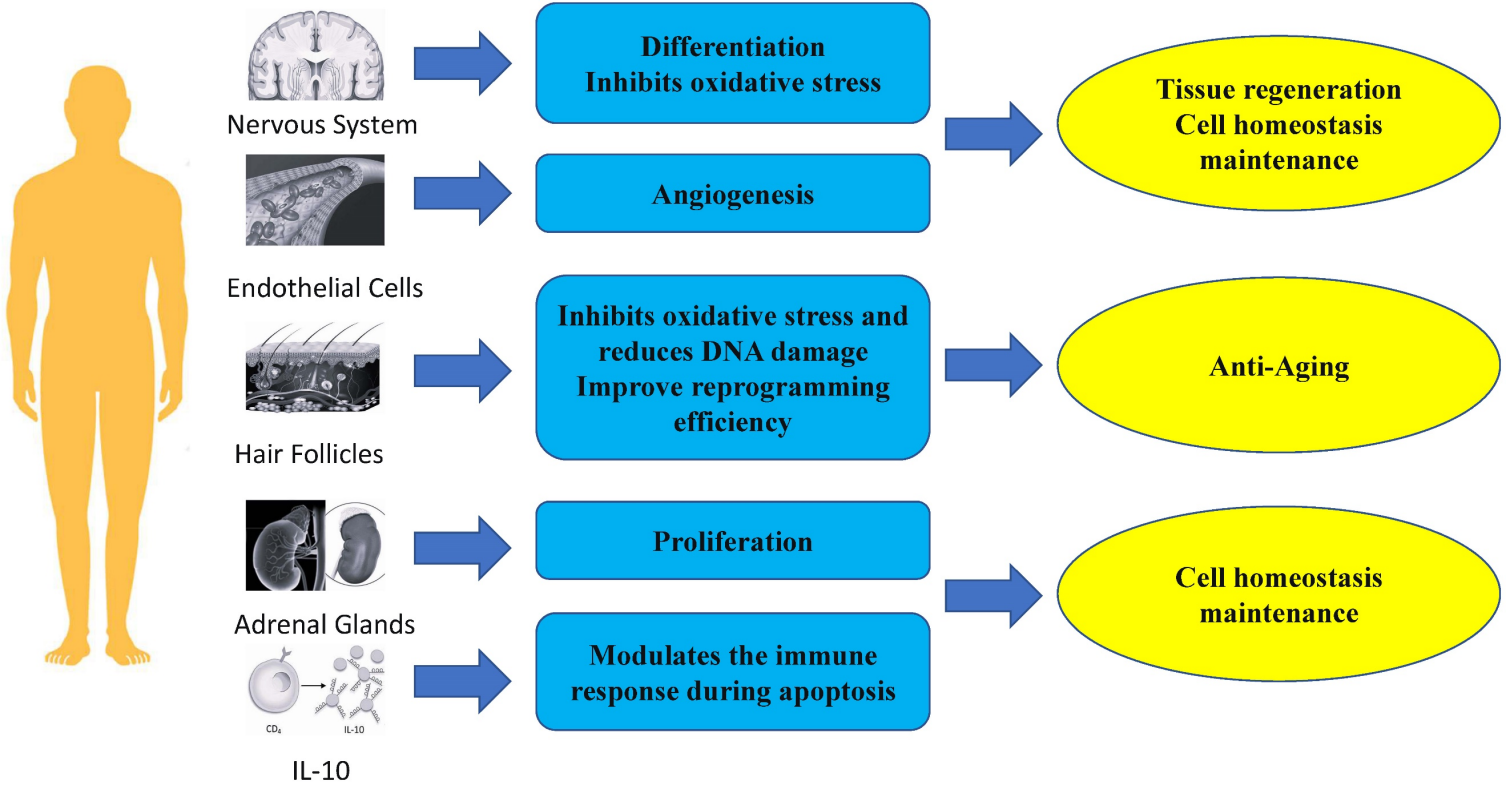
Role of Pbx1 in regenerative medicine.

In summary, PBX1 plays an important role in regenerative medicine, such as in anti-aging, maintenance of tissue homeostasis, and regeneration of tissues and organs (Fig. 2). Therefore, future clinical research on cell therapy and regenerative medicine with PBX1 modulation is warranted (FURGE2).

## Outlook

PBX1 lies at the intersection between cellular homeostasis and the regulation of responses to endogenous danger signals. In particular, PBX1 is a cellular factor that shows promise in the treatment of embryonic dysplasia, autoimmune diseases, diabetes, Alzheimer's disease, Parkinson's disease, trauma and other diseases, and there are potential targets for developmental and regenerative medicine research mechanisms. PBX1 is likely to become a hot spot in the treatment of other clinical diseases.

PBX1 has also been implicated in some neoplastic diseases, so there may be a risk of tumorigenesis in research on embryonic development and regenerative medicine. But further research is needed to be confirmed.

## Conclusion

PBX1 is a relatively new transcription factor that governs diverse cellular responses during normal development and tissue homeostasis. It possesses target site binding in closed chromatin, the ability to increase DNA access to other proteins, and is actively involved in cell fate specification. The mechanistic features of PBX1 in developmental and regenerative medicine may establish a link between the two pathways. PBX1 is a promising research target for future exploration of cellular homeostasis as well as the regulation of endogenous danger signals. It brings hope as a target therapy for a variety of diseases.

## Figures and Tables

**Figure 1:**
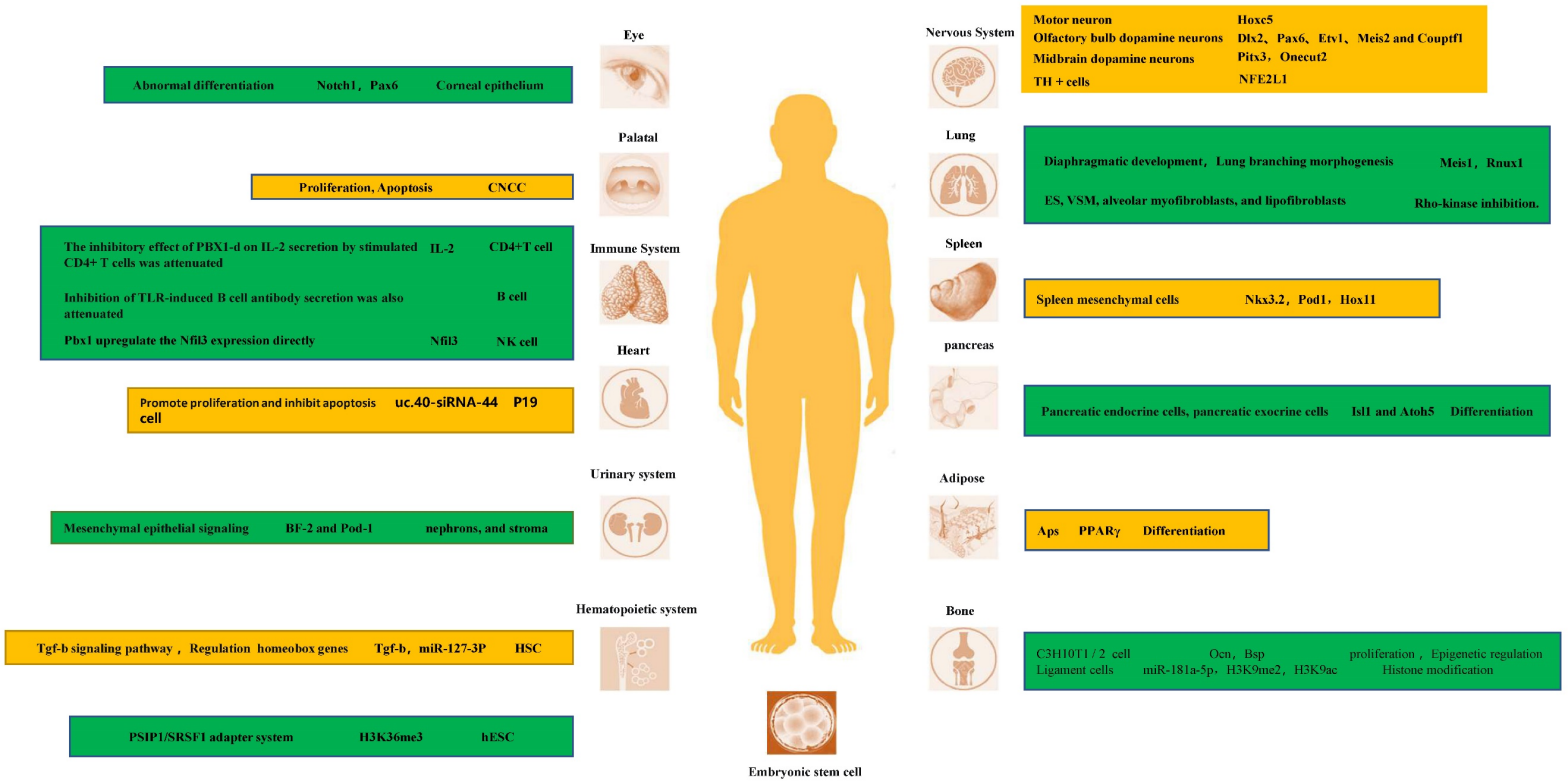
Research targets and underlying mechanisms of each system in development.

## Abbreviations

PBX1: Pre-B-cell leukemia transcription factor 1
HD: homeodomain
MN: motor neuron
ANS: Alternative splicing
TH: tyrosine hydroxylase
CNCC: Characterization of the cranial neural crest cell
ROS: reactive oxygen species
TAPs: transient amplifying progenitors
EC: endothelial cells
